# Neurotropism and interferon-dominated immune responses in a mouse-adapted coxsackievirus A16 infection model

**DOI:** 10.1128/jvi.00656-26

**Published:** 2026-06-30

**Authors:** Huijie Li, Rui Wang, Jichen Li, Wei Duan, Qiang Sun, Jianfang Zhou, Yong Zhang

**Affiliations:** 1 National Key Laboratory of Intelligent Tracking and Forecasting for Infectious Diseases (NITFID), National Institute for Viral Disease Control and Prevention, Chinese Center for Disease Control and Prevention 12415, Beijing, China; 2 National Laboratory for Poliomyelitis, WHO WPRO Regional Polio Reference Laboratory, National Institute for Viral Disease Control and Prevention, Chinese Center for Disease Control and Prevention 12415, Beijing, China; 3 National Health Commission Key Laboratory of Microbial Genomics, National Institute for Viral Disease Control and Prevention, Chinese Center for Disease Control and Prevention 12415, Beijing, China; 4 National Health Commission Key Laboratory for Biosafety, National Institute for Viral Disease Control and Prevention, Chinese Center for Disease Control and Prevention 12415, Beijing, China; University of Michigan Medical School, Ann Arbor, Michigan, USA

**Keywords:** Coxsackievirus A16, neonatal mouse model, neurovirulence, viral adaptation, interferon response

## Abstract

The development of animal models infected with Coxsackievirus A16 (CVA16) is limited by insufficient neurovirulence, which hinders the study of pathogenesis mechanisms and antiviral drug evaluation. Therefore, we aimed to develop a neonatal ICR mouse model for CVA16 infection, wherein infected brain tissue was continuously passaged from 2-day-old ICR suckling mice, resulting in a highly neurovirulent mouse-adapted strain (CVA16-P5) to establish optimal infection conditions for antiviral assessment. By systematically optimizing viral dose, inoculation route, and age at infection, we defined conditions that reproducibly induced progressive neurological disease and multisystem involvement. Infection with the CVA16-P5 strain resulted in consistent pathological alterations across multiple tissues, accompanied by characteristic neurological manifestations. Transcriptomic analysis of neural tissues revealed tissue-specific interferon-dominated immune responses and signatures of systemic viral dissemination that recapitulate key features of severe human infection. Whole-genome sequencing identified a nonsynonymous mutation in the VP1 capsid protein associated with enhanced neurovirulence during mouse adaptation. Overall, this CVA16-P5-adapted strain infection model provides a robust experimental platform for investigating CVA16 pathogenesis and for the preclinical evaluation of vaccines and antiviral drugs against neurotropic enteroviruses.

**IMPORTANCE:**

The lack of animal models that reliably recapitulate the neurological manifestations of Coxsackievirus A16 infection has constrained progress in understanding CVA16 neuropathogenesis. Here, we describe a neonatal mouse model based on a mouse-adapted CVA16 strain (CVA16-P5) that consistently induces neurological disease and multisystem pathology. This model enables the analysis of tissue-specific immune responses, viral dissemination, and genetic determinants of neurovirulence, including a VP1 mutation associated with enhanced pathogenicity. By providing a reproducible and physiologically relevant system for studying severe CVA16 infection, the CVA16-P5-adapted strain infection model setup in this study supports mechanistic studies of CVA16 pathogenesis and facilitates the preclinical evaluation of vaccines and antiviral drugs against neurotropic enteroviruses.

## INTRODUCTION

Coxsackievirus A16 (CVA16) is a major etiological agent of hand, foot, and mouth disease (HFMD) and represents a substantial public health burden, particularly among children aged under 5 years in the Asia-Pacific region (1, 2). Epidemiological surveillance data from multiple countries indicate that CVA16 consistently circulates alongside enterovirus A71 (EV-A71) and other enteroviruses, contributing to recurrent HFMD outbreaks with heterogeneous clinical outcomes (1, 2). Although historically, CVA16 has been less commonly associated with severe neurological disease compared to EV-A71, an increasing number of clinical reports indicate that CVA16 infection can be accompanied by severe neurological complications, including brainstem encephalitis, aseptic meningitis, and acute flaccid paralysis. Some cases also exhibit long-term neurological sequelae (3, 4). CVA16 molecular epidemiology reveals complex patterns of viral evolution that complicate intervention strategies. Genomic analyses demonstrated that CVA16 exhibits substantial genetic diversity, with multiple circulating genotypes and frequent intratypic recombination events (5). This genetic plasticity, particularly in the VP1 capsid region containing major neutralizing epitopes, presents a significant challenge for vaccine development (6, 7). Furthermore, co-circulation and co-infection with other enteroviruses may facilitate the emergence of novel recombinant strains with altered virulence properties (1, 5).

The lack of suitable animal models that accurately recapitulate key features of human disease has hindered investigations into the mechanisms of CVA16 infection. Current models primarily rely on transgenic mice and immunodeficient mice (8, 9). Transgenic mice expressing human viral entry receptors, such as scavenger receptor class B member 2 (SCARB_2_) and P-selectin glycoprotein ligand-1 (PSGL-1) transgenic mice, provide valuable platforms for studying virus-receptor interactions and host range restriction (10, 11). For example, the human SCARB_2_ knock-in mouse models support CVA16 infection and enable studies of viral replication and neurovirulence (10, 11). However, infections in these models are typically achieved via intracerebral inoculation, which bypasses the peripheral infection routes and may prioritize local brain pathology (11, 12). Consequently, these models fail to fully recapitulate the clinical manifestations of HFMD, particularly mucosal and cutaneous lesions and peripheral tissue involvement (1). Thus, there is a need for models that permit peripheral viral entry and subsequent dissemination to the central nervous system to facilitate studies on viral adaptation, neural invasion, and host responses under more physiological conditions (13, 14). Immunodeficient mouse models, such as AG129 and interferon alpha/beta receptor knockout (IFNAR^−/−^) mice, can also be infected with CVA16 during late development; however, their impaired immune systems limit their utility for studying the complete host immune response (8). More complex humanized mouse models have shown promise but are difficult to routinely implement due to their technical complexity and cost (9).

In contrast, neonatal mouse models, particularly ICR mice, are highly susceptible to enterovirus infection (8, 9). Infected mice consistently exhibit clinical symptoms, including limb and spinal cord paralysis, and high mortality under certain experimental conditions (15–17). These models have several advantages, including low cost, consistent viral loads, and disease progression, and their status as well-established infection models for other enteroviruses (8, 9). This has facilitated comparisons across studies (9). However, the variability in the effects of different enterovirus infections on ICR mice limits their application (9, 18). To date, EV-A71 and CVA6 have been well-documented to induce neurological disease in neonatal mouse models, whereas comparable studies focusing on CVA16 remain limited (16, 19–21). Although the neonatal ICR mouse model is promising for investigating early infection dynamics and pathological mechanisms, it has not been extensively adopted for CVA16 studies, particularly for studies addressing viral adaptation and virulence (21).

In this study, we evaluated the use of a neonatal ICR mouse model of CVA16 infection for antiviral assessment. By establishing a mouse infection model and conducting pathological assessments of infection in various tissues, this study reproduced several key features associated with severe CVA16 infection, including tissue-specific viral tropism, progressive neuropathology, and central nervous system-specific interferon activation (4, 5, 18). The model exhibited age-dependent disparities in mouse neurotoxicity, supporting its utility for experimental investigation (3, 21). Systematic transcriptomic analysis of neural tissues elucidated the immune responses that occur during infection, particularly the activation of interferon-related pathways (22, 23). Overall, given the absence of any immunocompetent animal system that recapitulates CVA16-induced neurological disease, our findings establish an experimental framework for investigating the pathological mechanisms of CVA16, evaluating potential therapeutic strategies (8, 9, 24), and expediting the development of novel therapeutic approaches.

## RESULTS

### Establishment of a neonatal ICR mouse model of CVA16 infection

**Fig 1:**
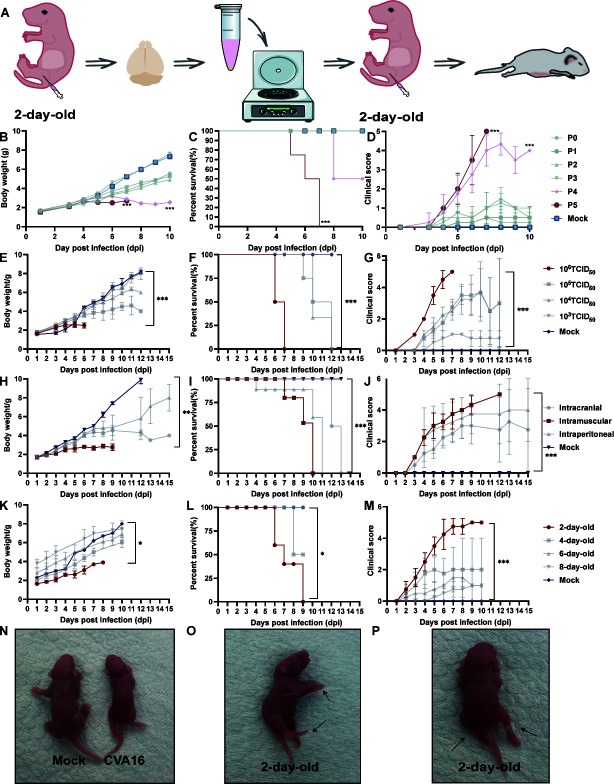
Establishment of a neonatal ICR mouse model infected with CVA16. (**A**) Schematic diagram of neonatal ICR mice adapting to CVA16 through continuous *in vivo* passage. (**B–D**) Passage generation: 2-day-old mice infected with CVA16 isolates from different passage generations, P0–P5, or mock-infected controls. (**E–G**) Dosage: 2-day-old mice with intramuscular inoculation of different dosages or mock-infected with CVA16-P5. (**H–J**) Route of inoculation: 2-day-old mice inoculated with 10^6^ TCID_50_ via three different routes of inoculation or mock-infected. (**K–M**) Age at inoculation: 2- to 8-day-old mice with intramuscular inoculation of 10^6^ TCID_50_ CVA16-P5 or mock-infected. (**B, E, H, and K**) Body weight. (**C, F, I, and L**) Percent survival. (**D, G, J, and M**) Scores. (N–P) Representative images of neonatal mice following CVA16 infection. Arrows indicate limbs exhibiting paralysis. Data are presented as mean ± standard deviation (SD). For body weight, survival, and clinical score analyses, *n* = 5 mice per group. *P < 0.05, **P < 0.01, ***P < 0.001.

By continuously passaging in newborn ICR mice, a mouse-adapted CVA16-P5 strain with enhanced neurovirulence was generated. After euthanizing 2-day-old ICR mice infected with the virus, brain tissue was collected, homogenized, and then inoculated into RD cells to amplify the virus. Then, the amplified virus supernatant was inoculated into naive 2-day-old ICR mice via intramuscular injection for the next passage. This procedure was repeated for five consecutive passages (P1–P5) (Fig. 1A).

In order to evaluate the phenotypic changes related to virus passage, the disease severity of mice infected with different passage viruses was compared. Compared with mice infected with viruses of lower passages, mice infected with viruses of higher passages exhibited more severe disease. In particular, P5 passage virus infection was associated with reduced body weight gain (Fig. 1B), decreased survival rates (Fig. 1C), and higher clinical scores (Fig. 1D), including earlier onset of limb weakness and paralysis.

To identify the most suitable infection conditions for model establishment, a dose-ranging experiment was performed. Serial dilutions of the P5 stock (dose range: 10⁶–10³ TCID_50_/50 μL) were intramuscularly injected into 2-day-old ICR mice, and body weight, survival, and clinical scores were monitored. At the highest viral dose (10⁶ TCID_50_/50 μL), infected mice showed rapid disease progression, characterized by limb weakness at 3 days post-infection (dpi), paralysis at 5–6 dpi, and complete mortality by 7 dpi. The intermediate doses (10⁵ and 10⁴ TCID_50_/50 μL) resulted in delayed disease onset, with clinical signs emerging around 7 dpi and partial mortality during the observation period. In contrast, the lowest dose that was tested (10³ TCID_50_/50 μL) caused only mild clinical manifestations without mortality (Fig. 1E through G). The animals in the mock control group exhibited no anomalies in weight gain or activity throughout the observation period. Based on these dose-response data, 10⁶ TCID_50_/50 μL was selected for subsequent experiments.

To compare infection outcomes associated with different inoculation routes, neonatal ICR mice were infected with CVA16 (10⁶ TCID₅₀/50 μL) via intraperitoneal, intracerebral, or intramuscular injection (Fig. 1H through J). Intraperitoneal inoculation resulted in variable disease progression, with one mouse succumbing at 4 dpi, and others exhibiting delayed mortality around 11 dpi. Intracerebral inoculation produced consistent disease outcomes, with all animals reaching terminal disease stages by 10 dpi. In contrast, intramuscular inoculation led to reproducible disease kinetics, with symptom onset at approximately 5 dpi and mortality occurring predominantly between 7 and 8 dpi. Based on the reproducibility of disease progression, intramuscular inoculation was selected for subsequent experiments.

Age-dependent susceptibility to CVA16 infection was evaluated by intramuscular inoculation of 10⁶ TCID_50_/50 μL virus into ICR mice at different postnatal ages (2, 4, 6, and 8 days) (Fig. 1K through M). Complete mortality was observed in 2-day-old neonatal mice by 8 dpi. In this group, body weight initially increased before declining, and clinical scores progressively worsened until death. In contrast, 4-day-old neonatal mice exhibited moderate susceptibility, with approximately 50% mortality by 9 dpi and milder clinical manifestations. Mice infected at 6 or 8 days of age showed transient weight loss followed by recovery, with no mortality. These results indicate a strong age-dependent susceptibility to CVA16 infection in neonatal ICR mice.

Neonatal mice infected at 2 days of age exhibited overt neurological manifestations, including reduced mobility, ataxia, lethargy, and flaccid limb paralysis (Fig. 1K through M). Distinct differences in posture and limb function were observed between infected and mock-treated animals (Fig. 1N). Both unilateral and bilateral limb paralysis were evident in infected mice (Fig. 1O and P).

### Systemic pathological manifestations in CVA16-infected mice

**Fig 2:**
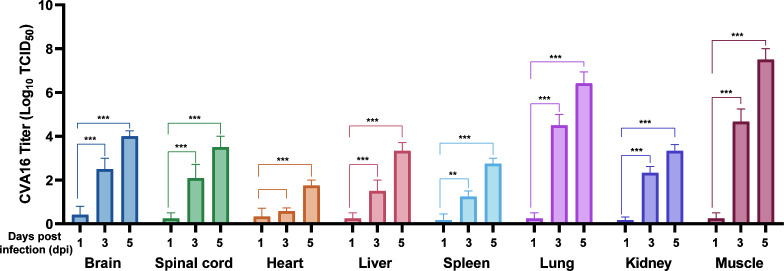
Virus titers in tissues of CVA16-infected neonatal mice. Two-day-old ICR mice were infected with CVA16 (10⁶ TCID_50_/50 μL) via intramuscular injection. Viral titers were determined in indicated tissues, including brain, spinal cord, heart, liver, spleen, lung, kidney, and muscle, at 1, 3, and 5 dpi. Data are presented as mean ± standard deviation (SD) (*n* = 3 mice per group). **P < 0.01, ***P < 0.001.

To characterize viral distribution following infection, viral titers were measured in multiple tissues at 1, 3, and 5 dpi (Fig. 2). Viral titers increased over time in all examined tissues, although the magnitude of viral accumulation differed among tissues. The highest viral titers were detected in skeletal muscle, followed by lung tissue. Detectable viral loads were also observed in brain and spinal cord, reaching approximately 10^4^ TCID_50_ at 5 dpi. Viral titers in the liver and kidney increased progressively during the course of infection.

**Fig 3:**
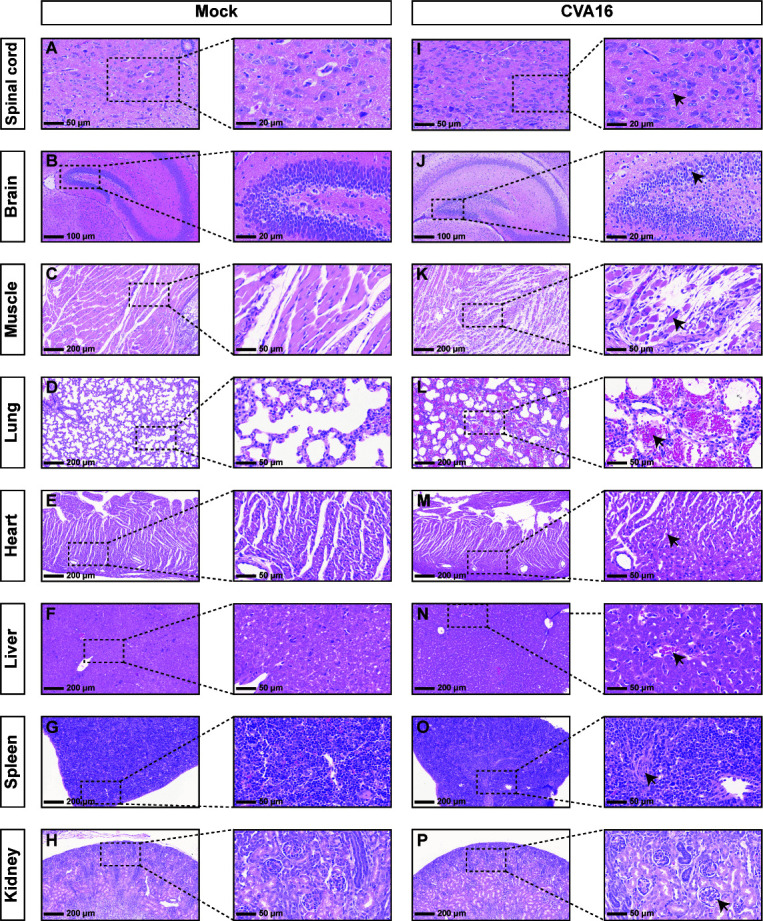
Histopathological analysis of multiple organs following CVA16 infection. Representative hematoxylin and eosin (H&E)-stained sections from mock-treated (left panels) and CVA16-infected (right panels) neonatal mice following intramuscular inoculation. Tissues examined include spinal cord (**A and I**), brain (**B and J**), muscle (**C and K**), lung (**D and L**), heart (**E and M**), liver (**F and N**), spleen (**G and O**), and kidney (**H and P**). Dashed boxes indicate the regions shown at higher magnification. Arrows indicate representative histopathological changes in CVA16-infected tissues, including neuronal vacuolation, hippocampal neuronal disorganization, skeletal muscle fiber disruption, alveolar septal thickening, myocardial fiber disorganization, hepatocellular degeneration, splenic architectural disruption, and glomerular structural alterations. Scale bars are indicated in each panel.

In order to further examine the histopathological changes associated with CVA16 infection, histopathological analysis was performed on multiple organs (Fig. 3). In the spinal cord (Fig. 3I), pathological changes were primarily observed in the ventral horn region, where neuronal vacuolation and disorganized neuronal arrangement compared with mock-infected controls (Fig. 3A). In the brain, CVA16-infected mice showed hippocampal neuronal disorganization, neuronal cell body atrophy, nuclear hyperchromasia, and decreased neuronal density (Fig. 3J), while hippocampal cytoarchitecture was preserved in the mock-infected controls (Fig. 3B).

Skeletal muscle exhibited pronounced pathological alterations following infection (Fig. 3K), including disruption of muscle fiber organization and cellular degeneration, while muscle tissue from mock-infected mice maintained normal morphology (Fig. 3C). Lung tissue from infected mice (Fig. 3L) showed thickened alveolar septa and intra-alveolar exudation compared with the intact alveolar architecture observed in mock-infected controls (Fig. 3D). In cardiac tissue, CVA16-infected mice exhibited myocardial fiber disorganization and focal inflammatory cell infiltration (Fig. 3M), while the myocardial structure remained intact in mock-infected controls (Fig. 3E).

In the liver, CVA16-infected mice exhibited hepatocellular degeneration and focal damage to liver structure (Fig. 3N), while the liver structure remained intact in mock-infected controls (Fig. 3F). In the spleen, disruption of normal splenic organization was observed in infected mice (Fig. 3O), while the mock group retained normal splenic architecture (Fig. 3G). Kidney sections from infected mice (Fig. 3P) showed structural alterations within the glomerular region relative to mock-infected controls (Fig. 3H).

To further support the representative histological observations, semi-quantitative histopathological scoring was performed for each examined tissue (Fig. S1). Compared with mock-infected controls, CVA16-infected mice showed significantly increased histopathological scores in the spinal cord, brain, skeletal muscle, lung, heart, and spleen, with the highest scores observed in skeletal muscle and lung tissues. In contrast, liver and kidney tissues showed relatively mild changes.

### Immunohistochemical manifestations in CVA16-infected mice

**Fig 4:**
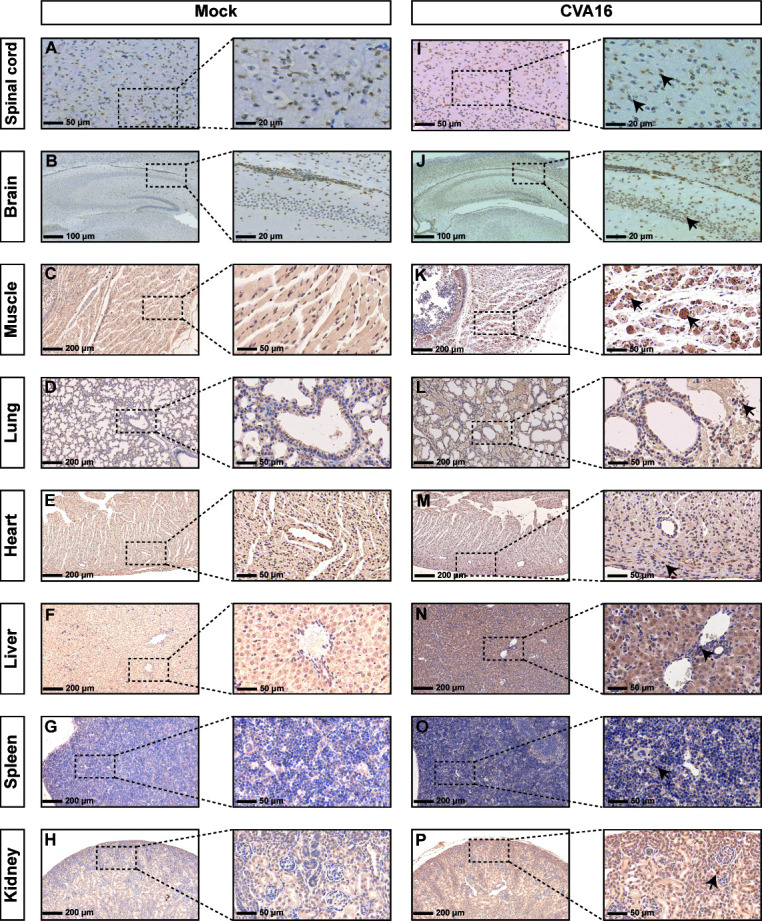
Immunohistochemical detection of CVA16 antigen in multiple organs of infected neonatal mice. Immunohistochemical staining was performed on tissue sections from CVA16-infected and mock-infected neonatal mice using anti-CVA16 immune serum. Representative images of spinal cord (**A and I**), brain (**B and J**), muscle (**C and K**), lung (**D and L**), heart (**E and M**), liver (**F and N**), spleen (**G and O**), and kidney (**H and P**) are shown. Dashed boxes indicate regions shown at higher magnification. Arrows indicate representative CVA16 immunoreactive signals in infected tissues. Scale bars are indicated in each panel.

Immunohistochemical staining was performed using CVA16-specific antiserum to evaluate the tissue distribution of CVA16 antigens (Fig. 4). CVA16 immunoreactivity was most consistently detected in skeletal muscle, while staining signals were weaker or limited in other tissues. No similar staining was observed in the mock-infected control tissues.

In the skeletal muscle, clear immunoreactive signals were observed within muscle fibers in infected mice (Fig. 4K), while no staining was detected in mock-infected controls (Fig. 4C). In lung sections, weak and limited immunoreactive signals were observed in discrete cellular regions of infected mice (Fig. 4L), whereas lung tissue from mock-infected mice showed no comparable staining (Fig. 4D).

Weak or localized immune response signals were observed in the spinal cord and brain tissues of infected mice, including cells around the central canal and adjacent gray matter areas in the spinal cord (Fig. 4I) and local regions of the hippocampus in the brain (Fig. 4J). No similar immunoreactivity was observed in the mock-infected controls (Fig. 4A and B).

In the liver, kidney, and cardiac tissues of infected mice, CVA16 immunoreactivity was weak, sparse, or limited to focal areas of infected mice (Fig. 4N, P, and M), while no similar staining was observed in the corresponding mock-infected tissues (Fig. 4F, H, and E). Combined with the viral titer data, these findings suggest that skeletal muscle was the major tissue consistent with viral antigen detection, and the main evidence of viral dissemination to other tissues was mainly supported by the results of tissue viral titers.

### Neurotropism and neuropathological changes induced by CVA16 infection

**Fig 5:**
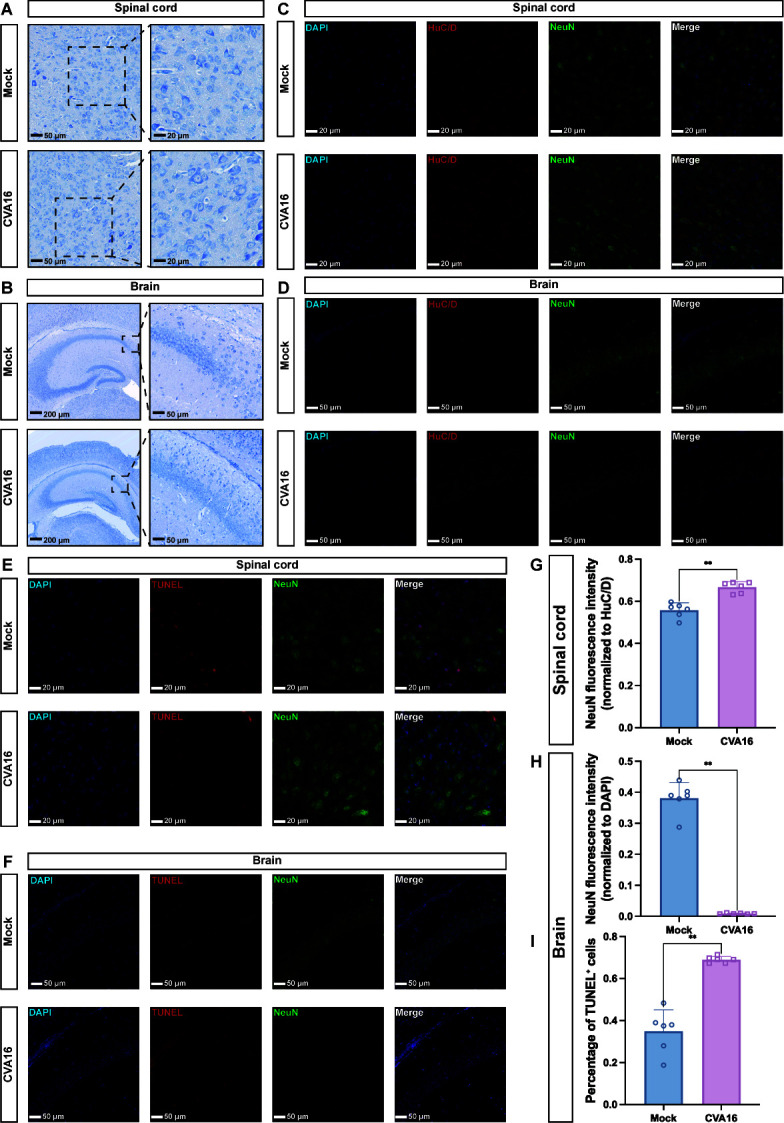
Neuronal marker expression and apoptosis in the spinal cord and brain following CVA16 infection. Histological and immunofluorescence analyses were performed on spinal cord and brain sections from CVA16-infected and mock-infected neonatal mice. (**A and B**) Representative images of Nissl-stained sections of the spinal cord (**A**) and brain (**B**). (**C and D**) Immunofluorescence staining of spinal cord (**C**) and brain (**D**) sections showing DAPI (blue), HuC/D (red), NeuN (green), and merged images in mock and CVA16-infected groups. (**E and F**) TUNEL staining combined with NeuN immunofluorescence in the spinal cord (**E**) and brain (**F**). DAPI (blue), TUNEL (red), NeuN (green), and merged images are shown. (**G**) Quantification of NeuN fluorescence intensity normalized to HuC/D in the spinal cord. (**H**) Quantification of NeuN fluorescence intensity normalized to DAPI in the brain. (**I**) Quantification of TUNEL-positive cells in the brain. The data are presented as mean ± standard deviation (SD) (*n* = 6 images per group). As shown, statistical significance was determined as indicated (**P < 0.01). Each panel is marked with a scale bar.

Histological analyses were performed on brain and spinal cord tissues from CVA16-infected mice to evaluate neuropathological changes (Fig. 5). Nissl staining of spinal cord sections showed a reduced number of Nissl-positive neurons in CVA16-infected mice compared with mock controls, which was further supported by quantitative analysis (Fig. 5A; Fig. S2A). Higher-magnification images further showed shrunken neuronal cell bodies and reduced Nissl substance staining within the affected spinal cord regions (Fig. 5A). Consistent with these findings, Nissl staining of brain sections demonstrated a decreased number of Nissl-positive neurons in CVA16-infected mice relative to mock-treated mice, which was confirmed by quantitative analysis (Fig. 5B; Fig. S2B).

To further characterize the changes in neurons, immunofluorescence staining of neuronal markers was performed. In spinal cord sections, HuC/D- and NeuN-positive signals were detected in both mock and infected groups; however, differences in signal intensity and distribution patterns were observed between the two groups (Fig. 5C). In brain sections, NeuN immunoreactivity in CVA16-infected mice was significantly lower than that in mock control mice (Fig. 5D).

Apoptotic cells were evaluated using TUNEL staining combined with NeuN immunofluorescence. In the spinal cord, TUNEL-positive signals were detected in both mock and CVA16-infected mice, and quantitative analysis showed no significant increase in the percentage of TUNEL-positive cells in infected mice compared with mock controls (Fig. 5E; Fig. S2C). In contrast, the number of TUNEL-positive cells in the brain tissues of CVA16-infected mice was increased compared with the mock control group (Fig. 5F and I). Merged images revealed partial spatial overlap between TUNEL-positive signals and NeuN-labeled cells in brain sections.

Quantitative analysis was performed based on fluorescence images. In the spinal cord, the NeuN fluorescence intensity of CVA16-infected mice was normalized to HuC/D and exhibited significant changes compared to the mock control group (Fig. 5G), indicating an altered neuronal marker profile. In the brain tissues of infected mice, the NeuN fluorescence intensity normalized to DAPI was significantly reduced (Fig. 5H). In addition, quantitative assessment showed that the percentage of TUNEL-positive cells in the brains of CVA16-infected mice was significantly higher than that in the mock-infected control group (Fig. 5I).

### Transcriptomic analyses revealed molecular changes in neural tissues following CVA16 infection

**Fig 6:**
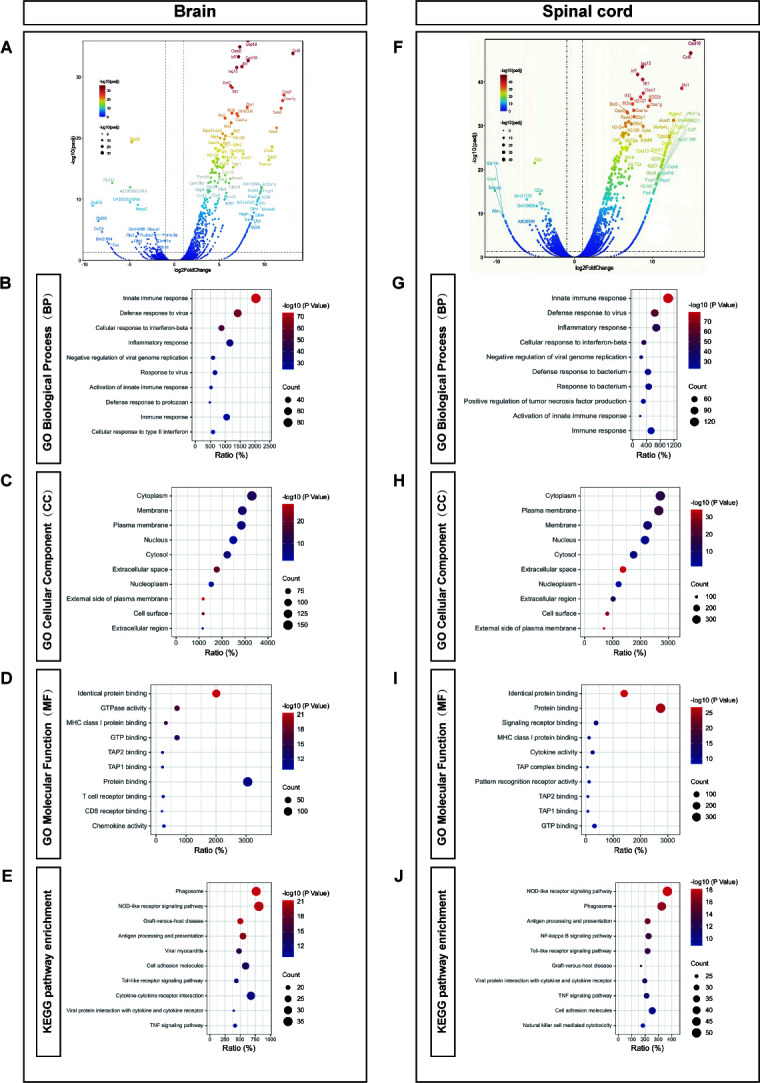
Transcriptomic analysis of brain and spinal cord tissues from CVA16-infected mice. (**A**) Volcano plot of differentially expressed genes (DEGs) in the brain of CVA16-infected mice compared with mock-infected controls. (**B–D**) Gene Ontology (GO) enrichment analysis of brain DEGs, including biological process (BP) (**B**), cellular component (CC) (**C**), and molecular function (MF) (**D**). (**E**) KEGG pathway enrichment analysis of brain DEGs. (**F**) Volcano plot of DEGs in the spinal cord of CVA16-infected mice compared with mock-infected controls. (**G–I**) Gene Ontology (GO) enrichment analysis of spinal cord DEGs, including BP (**G**), CC (**H**), and MF (**I**). (**J**) KEGG pathway enrichment analysis of spinal cord DEGs. Brain and spinal cord tissues were collected from CVA16-infected and mock mice for RNA sequencing, with *n* = 3 mice per group for each tissue.

Comprehensive RNA-seq analysis of brain and spinal cord tissues from CVA16-infected mice revealed widespread differential gene expression compared with mock-infected controls (Fig. 6A and F). Differentially expressed genes (DEGs) were identified using an adjusted *P*-value < 0.05 and |log_2_ fold change| > 1. In the spinal cord, 1,732 genes were upregulated, and 1,053 were downregulated, whereas in the brain, 1,241 genes were upregulated, and 987 were downregulated. The magnitude of gene expression changes was greater in the spinal cord, with a maximum log₂ fold change of 8.7 for upregulated genes, compared with 6.4 in the brain.

GO enrichment analysis of DEGs in the brain tissues revealed significant enrichment in biological processes related to immune and antiviral responses, including innate immune response, interferon-mediated signaling, and inflammatory responses (Fig. 6B). Cellular component analysis indicated that these genes were primarily associated with the cell membrane, cytoplasm, and extracellular regions (Fig. 6C). Molecular function analysis showed enrichment in immune-related activities, such as protein binding, major histocompatibility complex (MHC) binding, and GTPase activity (Fig. 6D). Kyoto Encyclopedia of Genes and Genomes (KEGG) pathway analysis further identified enrichment in pathways related to phagocytosis, NOD-like receptor signaling, antigen processing and presentation, and viral infection (Fig. 6E).

GO enrichment analysis of spinal cord tissues showed that DEGs were enriched in immune-related biological processes, particularly those involved in antiviral defense, inflammatory responses, and interferon-mediated signaling (Fig. 6G). Cellular component analysis revealed enrichment in the plasma membrane, cytoplasm, and extracellular regions (Fig. 6H). Molecular function analysis demonstrated enrichment in protein binding, MHC class I binding, and receptor recognition activity (Fig. 6I). KEGG pathway analysis identified significant enrichment in the NOD-like receptor signaling, phagocytosis, antigen processing and presentation, and viral infection-associated pathways (Fig. 6J).

### E241K mutation in the VP1 HI-loop domain correlates with increased virulence during mouse adaptation

Whole-genome sequencing of CVA16 isolates obtained after serial passage in neonatal mice (P0–P5) identified two nucleotide variations associated with viral adaptation (Table 1). One synonymous substitution was detected at nucleotide position 1717 within the VP3-coding region, which did not result in an amino acid change. The second variation was a non-synonymous mutation at nucleotide position 3616 in the VP1 gene, leading to a glutamic acid to lysine substitution at amino acid residue 241 (E241K).

**TABLE 1:** Nucleotide (Nt) and amino acid (Aa) differences in genomes between CVA16-P0 and CVA16-P5

Position	VP3	VP1
Nt/Aa	1717/1	3616/241
CVA16-P0	C/G	G/E
CVA16-P5	G/G	A/K

**Fig 7:**
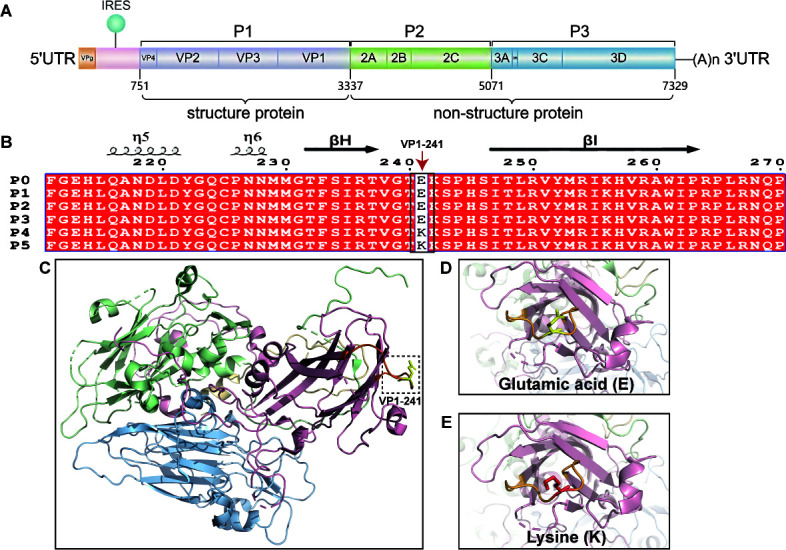
Identification of the VP1 E241K mutation during mouse adaptation. (**A**) A schematic diagram of the CVA16 genome structure, indicating the coding regions of structural protein (P1) and non-structural proteins (P2 and P3). (**B**) Amino acid sequence alignment of the VP1 protein from CVA16 isolates obtained after serial *in vivo* passages (P0–P5). The amino acid residue at position 241 within the HI-loop region is indicated. (**C**) Structural localization of residue 241 in the CVA16 capsid. VP1 is shown in pink, VP2 in blue, VP3 in green, and VP4 in light yellow. The HI-loop is highlighted in orange, and residue 241 in VP1 is indicated. (**D**) Enlarged view of glutamic acid at residue 241 in VP1. Glutamic acid is shown in yellow. (**E**) Enlarged view of lysine at residue 241 in VP1. The mutated lysine is shown in red. Structural images in panels C–E were generated using PyMOL version 3.0.3 based on the CVA16 capsid structure from the Protein Data Bank (PDB ID: 8X98).

The schematic analysis of CVA16 polyprotein indicated that this substitution (E241K) is located within the VP1 structural protein (Fig. 7A). VP1 sequence alignment of different passages (P0–P5) showed that the residue 241 is positioned in the HI-loop region, which was the surface-exposed region of the viral capsid (Fig. 7B). The E241K mutation was absent from P0 to P3, detected in P4, and retained in P5. Phenotypically, P4 infection caused partial mortality, whereas P5 infection resulted in complete mortality, indicating a progressively enhanced and more stable pathogenic phenotype during serial passage.

Structural modeling showed that residue 241 is located in the exposed HI-loop of VP1 (Fig. 7C). A detailed view further showed glutamic acid at this position in the parental virus (Fig. 7D), whereas this residue was replaced by lysine in the adapted virus (Fig. 7E).

### VP1 E241K substitution is associated with enhanced viral internalization, replication efficiency, and disease severity

**Fig 8:**
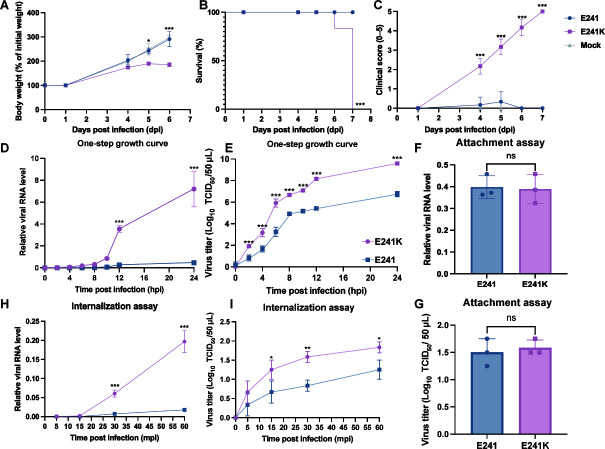
Characterization of viral entry, replication, and *in vivo* outcomes of CVA16 VP1 E241 and E241K variants. (**A–C**) *In vivo* outcomes of neonatal ICR mice infected with recombinant CVA16 variants carrying VP1 E241 or E241K, including body weight changes (**A**), survival curves (**B**), and clinical scores (**C**). (**D and E**) One-step growth curve analyses of recombinant CVA16 variants carrying VP1 E241 or E241K, evaluated by viral RNA quantification (**D**) and infectious virus titers measured by TCID₅₀ assays (**E**). (**F and G**) Viral attachment assays comparing recombinant CVA16 variants carrying VP1 E241 or E241K. Viral attachment was assessed by quantification of cell-associated viral RNA (**F**), and infectious titers were determined by TCID₅₀ assays (**G**). (**H and I**) Viral internalization assays showing intracellular viral RNA levels (**H**) and intracellular infectious virus titers (**I**) at the indicated time points post-infection. For *in vitro* experiments (**D–I**), data are presented as mean ± standard deviation (SD) from three independent experiments (*n* = 3). For animal experiments (**A–C**), *n* = 5 mice per group. Statistical significance is indicated as *P < 0.05, **P < 0.01, and ***P < 0.001; ns, not significant.

To further investigate the impact of the VP1 E241K substitution, we generated recombinant CVA16 viruses carrying the original residue (E241) or the mutated residue (E241K) and analyzed them both *in vitro* and *in vivo*. We first evaluated the impact of the E241K substitution in neonatal mice. Compared to mice infected with the E241 virus, mice infected with the E241K virus exhibited greater weight loss (Fig. 8A), reduced survival rate (Fig. 8B), and higher clinical scores (Fig. 8C), indicating increased disease severity.

We next compared viral replication kinetics by conducting a one-step growth curve analysis. Over time, cells infected with the E241K virus exhibited higher viral RNA levels than cells infected with the E241 virus (Fig. 8D). Simultaneously, the infectious viral titer produced by the E241K virus was consistently higher than that of the E241 virus, as determined by the TCID₅₀ assay (Fig. 8E).

We then evaluated the attachment of the viruses to host cells. No significant differences were observed between the E241 virus and the E241K virus in terms of cell-associated viral RNA levels after attachment (Fig. 8F) or infection titers measured by the TCID₅₀ assay (Fig. 8G), indicating that the attachment efficiency of these two viruses was comparable.

We subsequently evaluated the internalization process of the virus. At multiple time points after infection, intracellular viral RNA quantification results indicated that the level of intracellular viral RNA in cells infected with the E241K mutant virus was significantly higher than that in cells infected with the wild-type E241 virus (Fig. 8H). Consistently, infectious titers during the internalization stage were also higher in cells infected with the E241K mutant virus than in those infected with the E241 virus (Fig. 8I), indicating that the mutation affected the internalization stage.

## DISCUSSION

In this study, a neonatal mouse model of severe CVA16 infection was established in 2-day-old ICR mice using a mouse-adapted P5 strain. This model recapitulates key pathological features of severe human CVA16 infection, including progressive limb paralysis and fatal neurological outcomes (4, 14, 18). Intramuscular inoculation of 10⁶ TCID_50_/50 μL provides a consistent and reproducible disease course, allowing viral dissemination from peripheral tissues to the central nervous system while avoiding the artificial effects associated with direct intracranial injection. This approach improves the physiological relevance of the model and is consistent with the well-recognized age-dependent susceptibility observed in human infants (2, 3).

The mouse-adapted P5 strain acquired two mutations in structural proteins during serial *in vivo* passage, including a synonymous substitution in VP3 and a non-synonymous E241K substitution in VP1. Structural considerations suggest that the E241K mutation, located within an exposed loop region of VP1, may influence viral entry or early replication processes (7, 25). Importantly, the functional relevance of this substitution was directly examined using reverse genetics. Recombinant CVA16 viruses carrying the E241K mutation showed enhanced viral entry during the internalization stage and increased replication efficiency *in vitro*, without affecting viral attachment. In neonatal mice, the E241K virus caused more severe disease manifestations, including greater body weight loss, reduced survival, and higher clinical scores compared with the parental virus. Together, these findings demonstrate that the VP1 E241K substitution is strongly associated with increased virulence during mouse adaptation.

Histopathological and immunohistochemical analyses revealed extensive tissue involvement after CVA16 infection, with significant pathological changes observed in skeletal muscle, lung, and central nervous system tissues. Although immunohistochemical detection of viral antigens in neural tissues was relatively limited, the presence of viral RNA, neuropathological changes, and persistent clinical paralysis collectively supports active infection in the nervous system. The significant involvement of spinal cord tissues is consistent with the motor deficits observed in severe CVA16 patients (4, 21). In our study, spinal cord tissues showed abnormal neuronal marker changes after infection, suggesting that CVA16 infection altered the neuronal status of the spinal cord. Age-dependent susceptibility was restricted to neonatal ICR mice, suggesting that developmental factors beyond receptor expression may be involved in innate immune maturation or neural microenvironment properties and may be associated with disease severity (14, 26).

Transcriptomic analyses revealed a pronounced interferon-dominated immune signature in both brain and spinal cord tissues, with a greater magnitude and number of DEGs detected in the spinal cord. Enrichment of interferon-stimulated genes, innate immune pathways, and antigen presentation machinery highlights robust activation of antiviral defense mechanisms within neural tissues (22, 23). While these responses may contribute to viral restriction, excessive or sustained interferon signaling may also participate in immune-mediated neuropathology (23, 27, 28). Compared with the brain, the distinct immune response characteristics observed in the spinal cord may be related to the preferential involvement of motor pathways, which has also been reported in other neurotropic enterovirus infections such as poliovirus (26). In contrast, brain tissues showed a clearer reduction in neuronal marker signals together with increased apoptotic cells, indicating more evident neuronal loss and injury in the brain.

The findings of this study provide a new perspective on the CVA16 pathogenesis. It is worth noting that CVA16 exhibited severe neurovirulence in ICR mice only after five passages in ICR mice, indicating that CVA16 has rapid adaptive ability under selective pressure. The identification of the VP1-E241K substitution highlights that this residue is a potential hotspot for adaptive evolution and deserves further investigation of its role in viral adaptation and pathogenicity. In addition, the significant interferon response observed in spinal cord tissues emphasizes the dual role of innate immunity in neurotropic viral infections, serving as both a protective and potentially pathogenic factor, consistent with clinical observations of severe pediatric cases.

Although this model provides a standardized platform for studying neurovirulence and antiviral efficacy, it has some limitations. Infection could still occur in neonatal mice within the first 5 days after birth, but a stable lethal phenotype was achieved only in 2-day-old mice, which limits its broader applicability. In addition, intramuscular inoculation bypasses the natural mucosal route of enterovirus transmission. Although the model contributes to robust analysis of neurovirulence, its extreme disease severity may limit its use for vaccine evaluation. Further studies are needed on reverse genetics, longitudinal viral tracking, and immune cell profiling to elucidate the precise mechanisms of neuronal tropism and interferon-mediated immunopathology.

Compared with transgenic mouse models, such as human SCARB2 knock-in mice that support CVA16 infection after intracranial inoculation (10–12), our model is based on virus adaptation through continuous *in vivo* passage and peripheral muscle inoculation, allowing the virus to spread to the central nervous system under more physiological conditions. These two methods have complementary experimental purposes: the transgenic receptor knock-in model is highly suitable for evaluating different circulating CVA16 clinical isolates, while the standardized mouse-adapted strain described in this study provides a reproducible platform for antiviral screening and identification of viral determinants of neurotoxicity in a consistent host context.

In summary, this study established a reproducible neonatal mouse model of severe CVA16 infection with an immunocompetent host background and identified a key VP1 mutation associated with enhanced neurovirulence. This CVA16-P5-adapted strain infection model setup in this study provides a valuable experimental platform for understanding the pathogenesis of CVA16 and preclinical evaluation of vaccines and antiviral drugs against neurotropic enteroviruses.

## MATERIALS AND METHODS

### Cells and virus strains

#### Cells

Human rhabdomyosarcoma (RD) cells are derived from ATCC and numbered CCL-136. They were cultured in Dulbecco’s Modified Eagle’s Medium (DMEM) supplemented with 10% fetal bovine serum (FBS) and 1% penicillin-streptomycin at a temperature of 37°C and a carbon dioxide concentration of 5%.

#### Virus

The CVA16 strain HB10-114 was isolated from a throat swab sample of a mild hand-foot-mouth disease patient in Hebei Province, China, who developed symptoms on May 12, 2010. It was propagated in RD cells. When the degree of cell fusion reached 80%–90%, the medium was replaced with a medium containing 2% FBS, and the cells were incubated with the virus to observe a cytopathic effect. After three freeze-thaw cycles, all viruses in the supernatant were filtered through a 0.22-μm filter and stored at −80°C.

### CVA16 full-genome sequencing

Whole-genome sequencing of CVA16 was performed by combining reverse transcription polymerase chain reaction (RT-PCR) and Sanger sequencing. Viral RNA was extracted from the infected cell cultures or clinical samples using the Tianlong Nucleic Acid Extraction System (Tianlong, China). The extracted RNA was reverse transcribed into cDNA using the Takara RT-PCR Kit (RR057A, Takara, Japan).

To obtain the complete CVA16 genome, a set of CVA16-specific primers was used to amplify overlapping genomic fragments spanning the entire genome. The primer sequences used for amplification are provided in Table S1. PCR amplification was performed using these primers under standard cycling conditions, and the purified PCR products were subjected to Sanger sequencing (29). The raw sequence data were assembled into a full-length genome sequence using Sequencher software (version 5.4.6; Gene Codes Corporation, Ann Arbor, MI, USA) to obtain the complete genome sequences of the CVA16 strains.

### Establishment of an ICR mouse model of CVA16 infection

To establish a CVA16 strain adapted to mice, 2-day-old ICR mice were injected with the CVA16-P0 strain (HB10-114) into the right hind limb muscle. The same volume of the virus-free maintenance medium for virus dilution was injected into the same site as mock-infected mice. Clinical symptoms exhibited by the mice, including weight loss, limb weakness, and paralysis, were monitored daily. In instances where severe symptoms were observed (clinical score of 4), brain tissue was collected and homogenized in phosphate-buffered saline (PBS). Subsequently, the tissue was centrifuged at 12,000 × *g* for 15 min. Next, the tissue was filtered through a 0.22-μm membrane to obtain a viral suspension for subsequent passage. After five consecutive passages in 2-day-old ICR mice, the adapted strain consistently induced paralysis within 72 h in the same mice. The final CVA16-P5 strain was obtained from brain tissue, amplified (using the same processing method), aliquoted, and stored at −80°C. The Reed-Muench method was used to determine viral titer in RD cells, yielding a titer of 10⁶ TCID_50_/50 μL. Genetic stability of viral samples was monitored throughout serial passaging throughout the process by sequencing the VP1 gene to ensure result quality (30). All *in vivo* infection experiments were performed using biologically independent neonatal ICR mice. Unless otherwise indicated, each experimental group consisted of five mice, and all experiments were repeated at least twice to ensure reproducibility.

### Tissue viral titer determination through TCID_50_ assay

Tissues were weighed and homogenized in PBS using a tissue grinder. Subsequently, centrifugation was performed (12,000 × *g*, 15 min, 4°C), and the samples were filtered (0.22 μm). Next, the sample was serially diluted (10^−1^–10^−9^) in DMEM containing 2% FBS, after which it was inoculated onto monolayers of RD cells in 96-well plates (50 μL/well, four wells per dilution). After a 72-h incubation period at 37°C in an atmosphere containing 5% carbon dioxide, the cytopathic effect was evaluated microscopically. Wells with >50% round cells were designated as positive. Viral titers were calculated using the Reed-Muench method, with the results expressed as –log_10_ TCID_50_ tissue (mean ± standard deviation of triplicate experiments).

### Histopathology and immunohistochemistry

Tissue samples were fixed with 4% paraformaldehyde, embedded in paraffin, and sectioned (5 µm). For skeletal muscle histopathology, samples were collected from the left hind limb muscle on the opposite side of the injection site to avoid tissue changes caused by direct needle puncture. The sections were stained with hematoxylin and eosin to assess inflammation, necrosis, and viral cytopathic effects. Viral antigen detection was performed using the in-house-prepared CVA16-immunized mouse antiserum as the first antibody at a dilution of 1:200. The antiserum was produced by immunizing BALB/c mice with purified mouse-adapted CVA16 particles at weeks 0, 2, and 4. After the final immunization, blood was collected from the orbital sinus and centrifuged to obtain serum. The collected serum was used for immunohistochemical staining and then incubated with horseradish peroxidase-labeled secondary antibody and subjected to DAB staining.

Based on the severity of tissue damage, inflammatory cell infiltration, cell degeneration, and structural destruction, tissue pathological damage was semi-quantitatively scored using a blinded method. The scoring criteria were as follows: 0, no obvious lesion; 1, minimal lesions; 2, mild lesions; 3, moderate lesions; and 4, severe lesions. For each tissue, three microscopic fields were randomly selected for evaluation. All images were encoded before scoring to ensure blinded evaluation. The average score of two independent observers was taken for statistical analysis.

### Nissl staining

Paraffin-embedded brain and spinal cord tissues were sectioned at a thickness of 5 μm. After deparaffinization and rehydration through graded ethanol, sections were stained with 0.1% crystal violet solution for Nissl staining. Excess dye was removed by brief differentiation, followed by dehydration, clearing, and mounting. Stained sections were examined under a light microscope. Neuronal morphology and Nissl substance distribution were examined by light microscopy. For Nissl staining quantification, Nissl-positive neurons were counted from three randomly selected microscopic fields from each mouse.

### Immunofluorescence and TUNEL assay

The tissue sections embedded in paraffin were deparaffinized, rehydrated, and subjected to antigen retrieval. After permeabilization and blocking, the sections were incubated overnight at 4°C with primary antibodies, including mouse anti-NeuN (ab177487, RBFOX3, Abcam, 1:200) and mouse anti-HuC/D (ab176106, ELAVL3/4, Abcam, 1:200). For viral antigen detection, CVA16-immunized mouse serum was used as described above.

After washing, the sections were incubated at 20°C with Alexa Fluor 488-conjugated goat anti-mouse IgG (Abcam, ab150113) or Alexa Fluor 594-conjugated goat anti-mouse IgG (Abcam, ab150116) for 1 h. Nuclei were counterstained with DAPI (Thermo Fisher Scientific, D1306). Apoptotic cells were detected using a TUNEL fluorescence assay kit (Solarbio, SF555) according to the manufacturer’s instructions. Fluorescence images were acquired using a Leica DMI8 fluorescence microscope under identical acquisition settings for all groups.

For immunofluorescence-based quantitative analyses, fluorescence intensity and TUNEL-positive cell percentages were quantified using Fiji (ImageJ, NIH, USA). Quantitative analyses were performed using three biologically independent mice per group, with three randomly selected microscopic fields evaluated for each mouse.

### RNA-seq and bioinformatics analysis

Total RNA was extracted from tissue samples using TRIzol reagent (Invitrogen, Thermo Fisher Scientific, USA) following the manufacturer’s instructions. The quality and integrity of the isolated RNA were verified prior to subsequent library construction. Transcriptome sequencing was subsequently performed. RNA library preparation and high-throughput sequencing were performed by Novogene Co., Ltd. (Beijing, China) in accordance with their standard operational procedures. Briefly, sequencing libraries were generated using qualified total RNA, and the constructed libraries were then sequenced on the Illumina NovaSeq 6000 platform to generate high-quality raw reads.

Raw sequencing reads were subjected to quality control and filtering, followed by alignment to the reference genome using standard bioinformatic pipelines. Differential gene expression analysis was performed using DESeq2 (v1.30.1) implemented in the R software package (30). Genes with an adjusted *P*-value < 0.05 and |log₂ fold change| > 1 were considered differentially expressed genes. To further explore the biological implications of these DEGs, GO and KEGG pathway enrichment analyses were performed using the DAVID Bioinformatics Resources (version 6.8) (31).

### Structural modeling of VP1 E241K

Structural localization of VP1 residue 241 was analyzed using the CVA16 capsid structure from the Protein Data Bank (PDB ID: 8 X 98). Structural visualization and residue highlighting were performed using PyMOL version 3.0.3. VP1, VP2, VP3, VP4, HI-loop, and residue 241 were colored and annotated for figure preparation.

### Reverse genetics and generation of recombinant CVA16 viruses

The full-length infectious cDNA clone of CVA16 strain HB10-114 was constructed based on the parental viral genome sequence. Viral genomic RNA was extracted from infected RD cells and reverse-transcribed into cDNA. The full-length CVA16 genome was amplified as overlapping fragments and assembled into a plasmid vector downstream of a T7 RNA polymerase promoter.

Site-directed mutagenesis was performed to introduce the E241K substitution into the VP1-coding region using a PCR-based mutagenesis strategy. All constructs were verified by Sanger sequencing to confirm the presence of the desired mutation and the absence of unintended nucleotide changes.

For the recovery of recombinant viruses, plasmids containing the full-length CVA16 cDNA (E241 or E241K) were linearized and used as templates for *in vitro* transcription with T7 RNA polymerase to generate infectious viral RNA. The synthesized RNA was transfected into RD cells using Lipofectamine 3000 (Thermo Fisher Scientific, USA) according to the manufacturer’s instructions. Following transfection, cells were monitored daily for cytopathic effects. Culture supernatants were collected upon extensive cytopathic effect development, clarified by centrifugation, and further amplified in RD cells. Recombinant viruses were aliquoted, stored at −80°C, and viral titers were determined by TCID_50_ assays prior to use in subsequent experiments (32).

### Virus attachment and internalization assays

To assess viral attachment, RD cells were pre-chilled at 4°C for 30 min and incubated with recombinant CVA16 viruses carrying VP1 E241 or E241K at a multiplicity of infection (MOI) of 5 at 4°C for 1 h to allow viral binding without internalization. Cells were subsequently washed extensively with cold PBS to remove unbound virus. Cell-associated viral RNA was extracted for quantitative RT-PCR analysis, or cells were harvested for TCID_50_ assays to determine attached infectious viral particles.

For viral internalization assays, after virus binding at 4°C for 1 h, cells were shifted to 37°C to allow viral internalization. At the indicated time points, cells were treated with acidic PBS (pH 3.0) to remove non-internalized surface-bound virus, followed by neutralization and washing. Intracellular viral RNA levels were quantified by qRT-PCR, and intracellular infectious titers were determined by TCID_50_ assays.

### One-step growth curve analysis

RD cells were infected with recombinant CVA16 viruses carrying VP1 E241 or E241K at an MOI of 5. After 1 h of adsorption at 37°C, cells were washed to remove unbound virus and incubated in fresh medium containing 2% FBS. At the indicated time points post-infection, cell culture supernatants and cells were collected. Viral replication was evaluated by quantifying viral RNA levels using qRT-PCR and by determining infectious virus titers using TCID_50_ assays.

### TCID_50_ assay for cell culture samples

Cell culture supernatants or cell lysates were collected at the indicated time points and clarified by centrifugation at 12,000 × *g* for 10 min at 4°C. Samples were serially diluted (10⁻¹–10⁻⁹) in DMEM containing 2% FBS and inoculated onto RD cell monolayers in 96-well plates (50 μL per well, four wells per dilution). After incubation for 72 h at 37°C with 5% CO₂, cytopathic effects were assessed microscopically. Viral titers were calculated using the Reed-Muench method and expressed as log₁₀ TCID_50_/50 μL.

### Quantitative RT-PCR analysis

Viral RNA was extracted from infected cells using a commercial RNA extraction kit according to the manufacturer’s instructions. Quantitative RT-PCR was performed using a fluorescence probe-based (TaqMan) method. Reverse transcription and amplification were carried out using a one-step qRT-PCR kit (RR064A, Takara, Japan) on a QuantStudio 5 Real-Time PCR System (Thermo Fisher Scientific, Waltham, MA, USA). CVA16-specific primers and a fluorescent probe targeting the VP1 region were used to quantify viral RNA levels. GAPDH was used as an internal control. Relative viral RNA levels were calculated using the comparative Ct method. All reactions were performed in triplicate.

Primer and probe sequences used for quantitative RT-PCR were as follows:

CVA16 VP1 forward primer: 5′-GCTGCTGCTTGTTCTTGTTG-3′

CVA16 VP1 reverse primer: 5′-CGAGTTGAGTTGCTGGTGTT-3′

CVA16 VP1 probe: 5′-FAM-AGCCAGTGTTGGAGGAGGAG-BHQ1-3′

GAPDH forward primer: 5′-AGGTCGGTGTGAACGGATTTG-3′

GAPDH reverse primer: 5′-TGTAGACCATGTAGTTGAGGTCA-3′

### Statistical analysis

Unless otherwise indicated, “n” refers to the number of biologically independent samples. For *in vivo* experiments, “n” indicates the number of individual mice per group. For RNA-seq analyses, “n” indicates the number of independently collected tissue samples per group. For image-based quantitative analyses, “n” represents the number of independently analyzed microscopic fields derived from at least three biologically independent animals per group.

Data are presented as mean ± standard deviation (SD) unless otherwise indicated. Comparisons between two groups were performed using a two-tailed Student’s *t*-test. For multiple group comparisons, one-way analysis of variance (ANOVA) followed by Dunnett’s multiple comparison test was used. Data not conforming to a normal distribution or exhibiting unequal variances were presented as the mean and 95% CI, and comparisons were performed using the Mann-Whitney U test. A *P*-value < 0.05 was considered statistically significant. Statistical significance is indicated as *P < 0.05, **P < 0.01, and ***P < 0.001, and “ns” denotes no significant difference.

Image analyses were performed using Fiji (ImageJ, NIH, USA), and statistical analyses were conducted using GraphPad Prism version 10. Figures were assembled using Adobe Illustrator 2021.

## Data Availability

The complete genome sequences of CVA16-P0 and CVA16-P5 have been deposited in the National Microbiology Data Center (NMDC), with the accession numbers NMDCN00097Q5 and NMDCN00097Q6, respectively.

## SUPPLEMENTAL MATERIAL

Figure S1 jvi.00656-26-s0001.tif Semi-quantitative histopathological scoring of multiple tissues following CVA16 infection.

Figure S2 jvi.00656-26-s0002.tif Quantification of Nissl-positive neurons and TUNEL-positive cells in neural tissues.

Supplemental material jvi.00656-26-s0003.docx Legends for Fig. S1 and S2; Table S1, primers used for amplification of overlapping CVA16 genomic fragments.

ASM does not own the copyrights to Supplemental Material that may be linked to, or accessed through, an article. The authors have granted ASM a non-exclusive, world-wide license to publish the Supplemental Material files. Please contact the corresponding author directly for reuse.
