# Supplementary figures and images for: Neurotropism and interferon-dominated immune responses in a mouse-adapted coxsackievirus A16 infection model
Source: J Virol. 2026 Jun 30;100(7):e00656-26. doi: 10.1128/jvi.00656-26 (PMC13386987; doi:10.1128/jvi.00656-26)

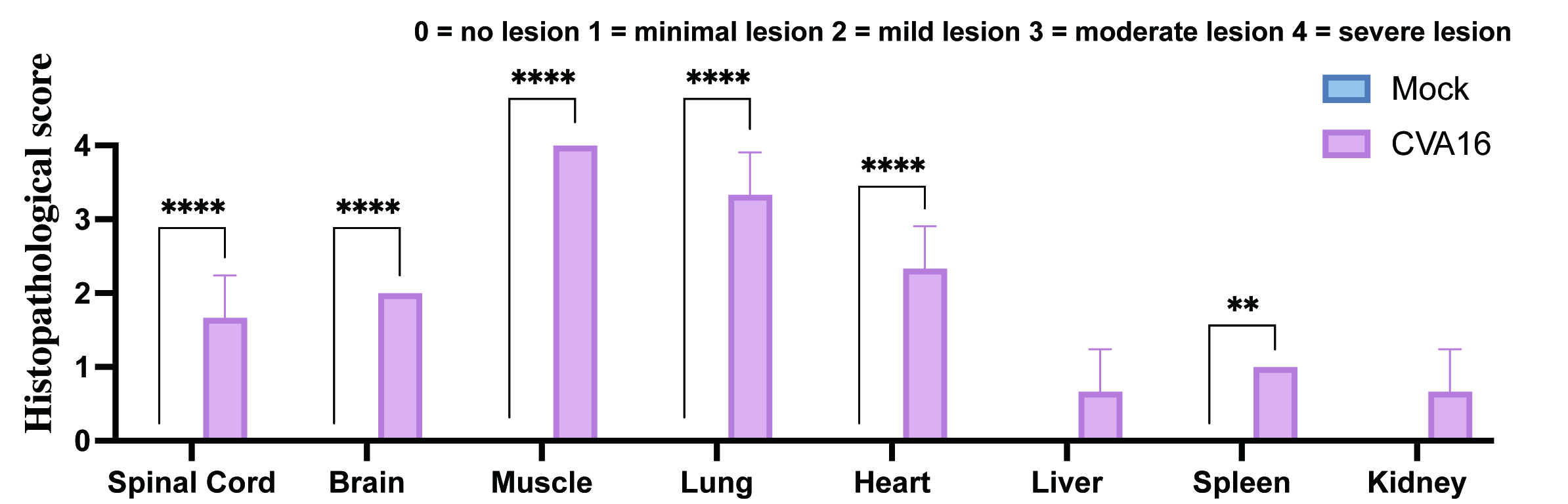

Supplement: Figure S1 — Semi-quantitative histopathological scoring of multiple tissues following CVA16 infection. [file jvi.00656-26-s0001.tif]

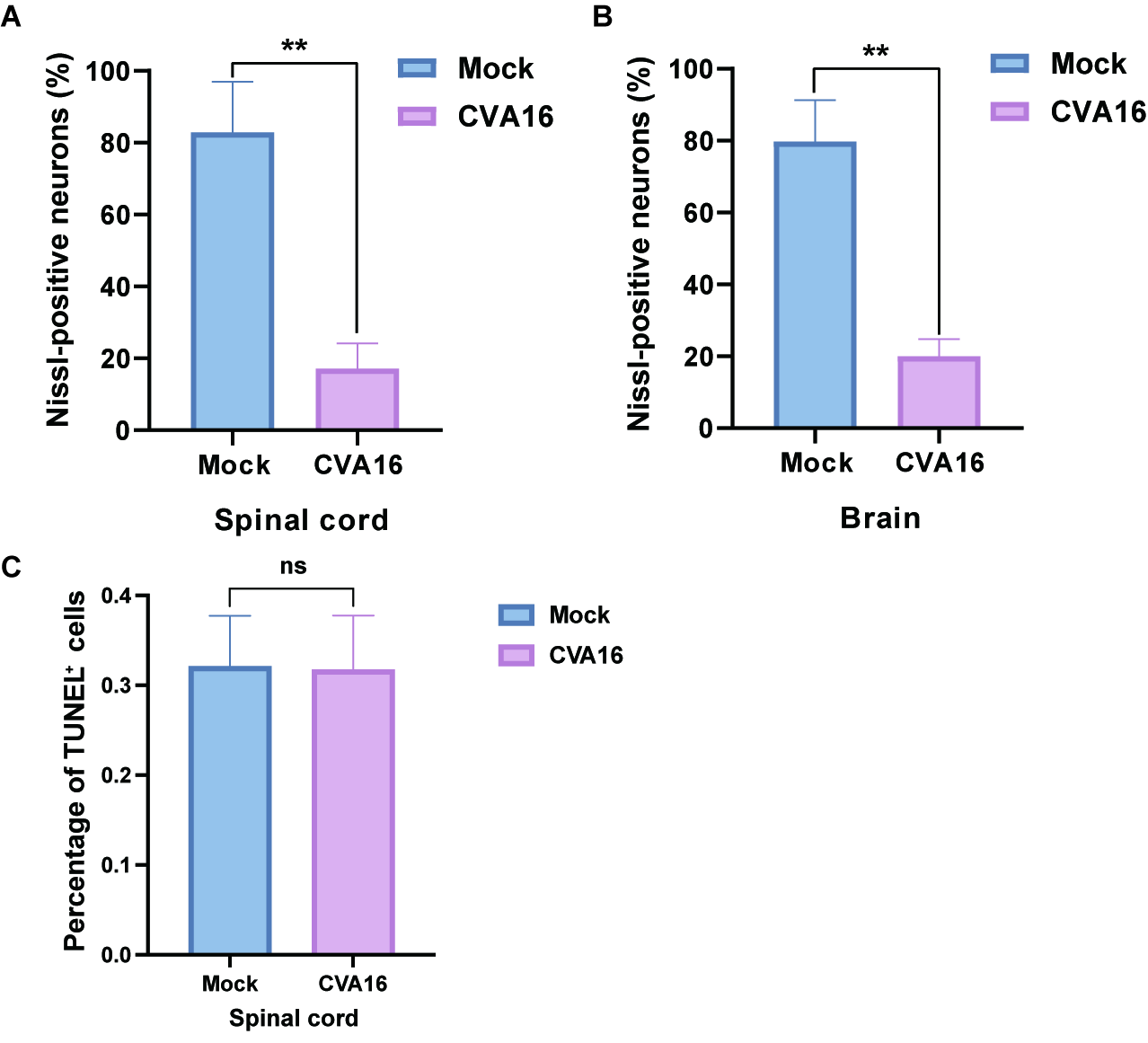

Supplement: Figure S2 — Quantification of Nissl-positive neurons and TUNEL-positive cells in neural tissues. [file jvi.00656-26-s0002.tif]
