# Supplementary material for: Neurotropism and interferon-dominated immune responses in a mouse-adapted coxsackievirus A16 infection model
Source: J Virol. 2026 Jun 30;100(7):e00656-26. doi: 10.1128/jvi.00656-26 (PMC13386987; doi:10.1128/jvi.00656-26)
Supplement: Supplemental material — Legends for Fig. S1 and S2; Table S1, primers used for amplification of overlapping CVA16 genomic fragments. [file jvi.00656-26-s0003.docx]

**Figure Legends**

**Supplementary Figure 1. Semi-quantitative histopathological scoring of multiple tissues following CVA16 infection.**

Semi-quantitative histopathological scores of indicated tissues from mock-treated and CVA16-infected mice. Scores were assigned based on the severity of tissue damage, inflammatory cell infiltration, cellular degeneration, and structural disruption. Data are presented as mean ± standard deviation (SD); n = 3 mice per group, with three randomly selected microscopic fields evaluated for each mouse. Statistical significance is indicated as ***P* < 0.01 and ****P* < 0.001.

**Supplementary Figure 2. Quantification of Nissl-positive neurons and TUNEL-positive cells in neural tissues.**

(A) Quantification of Nissl-positive neurons in the spinal cord. (B) Quantification of Nissl-positive neurons in the brain. (C) Quantification of TUNEL-positive cells in the spinal cord. Data are presented as mean ± standard deviation (SD); n = 3 mice per group, with three randomly selected microscopic fields evaluated for each mouse. Statistical significance was determined using the Mann-Whitney U test. ***P* < 0.01; ns, not significant.

**Supplementary Table 1. Primers used for amplification of overlapping CVA16 genomic fragments.**

| **Primer name** | **Sequence (5′ → 3′)** | **Length (nt)** | |
| --- | --- | --- | --- |
| CVA16-1  CVA16-2S  CVA16-2A  CVA16-3S  CVA16-3A  CVA16-4S  CVA16-4A  CVA16-5S  CVA16-5A  CVA16-6S  CVA16-6A  CVA16-7  CVA16-8 | AATTTCCAATACCAACCCTTTGA  GAGGCATGTGGTTACAGTGA  GACGCCCCTGTCTCCGCGGC  TGGTTTATGATGGTATCC  AAATATGACACATTACCAAACA  ATCCATGCCCAAGGGCTTCCAT  TGCATCACCAAACTGCTCT  GACAGGAAGTCTAAAGTGAGA  GGGTACCCGGCACTGGTGTG  AACAAGGAGAGATCCAATGG  CTGATTATTAGTTATTGATCAT  TATGATGCAAGTCTCAGCCC  GAAACACGGACACCCAAAGTAGT | | 23  20  20  20  22  22  20  21  20  20  23  20  23 |
